# Manual of Static Electricity in X-Ray and Therapeutic Uses

**Published:** 1897-08

**Authors:** 


### Book Reviews.

MANUAL OF STATIC ELECTRICITY IN X-RAY AND THERAPEUTIC USES. By S. H. MONELL, M. D., Founder and Chief Instructor of the Brooklyn Post-Graduate School of Clinical Electro-Therapeutics and Röntgen Photography ; Fellow of the New York Academy of Medicine. Octavo, 614 pages, cloth, gilt. Illustrated. New York : William Beverly Harison, Publisher, 3 and 5 W. 18th street. 1897.

This volume is the only exhaustive work so far published on the application and benefits to be derived from static electricity. The writer, who is a most ardent believer, has gone over the grounds admirably well and has championed the cause to the utmost. The adaptation of the X-ray to the Holtz apparatus enhances the value of this method of therapeutics and will encourage a wider field of usefulness.

The author divides the work in two parts, part first being devoted to a careful explanation of the apparatus, care of the Holtz machine and therapeutics of static electricity. The chapter on pain discloses the exactness of known indications for special methods of treatment. The chapter on morbid mental states is one of the most instructive and valuable in the book. Rheumatoid arthritis receives especially full consideration and directions for treatment of every stage are definitely described.

Two entire chapters establish the value of static electricity in the treatment of important skin diseases. These chapters will be found especially valuable to the general practitioner as well as to the dermatologist. The section devoted to the employment of static electricity in gynecology and the various phases of women's diseases states when and how to use this agent with this class of cases. The physiological properties of static electricity constitute the rational basis of its therapeutic uses and are fully stated.

Five chapters are devoted to Crookes' tubes, X-ray operative methods and X-ray photography. These chapters are original and contain complete practical instruction, much of which is not in print elsewhere. The directions are so plainly written out that a beginner can follow them successfully.

The second part of the book reviews the literature bearing upon static electricity, with quite an extended report from Grey's Hospital, where it was successfully used by Golding Bird, Mr. Hughes and Sir William Gull.

To those interested in electricity this volume is to be recommended, but it must always be borne in mind "that the best instrument only does the best work under the guidance of the best hand." The book is nicely printed and well bound. W. C. K.

